# A novel indirubin- 3-monoxime derivative I3MV- 8b exhibits remarkable cytotoxicity against multiple myeloma by targeting TRIM28

**DOI:** 10.1186/s40364-025-00773-3

**Published:** 2025-04-07

**Authors:** Teng Fang, Lanting Liu, Hao Sun, Xiaoyu Zhang, Xiyue Sun, Zhen Yu, Lixin Gong, Shiyi Xie, Yonglong Zhao, Yan Li, Lugui Qiu, Gang An, Bin He, Mu Hao

**Affiliations:** 1 State Key Laboratory of Experimental Hematology, National Clinical Research Center for Blood Diseases, Haihe Laboratory of Cell Ecosystem, Institute of Hematology & Blood Diseases Hospital, Chinese Academy of Medical Sciences & Peking Union Medical College, Tianjin, 300020 China; 2 Department of Hospital Management, Gobroad Healthcare Group, Beijing, China; 3 State Key Laboratory of Functions and Applications of Medicinal Plants, Engineering Research Center for the Development and Application of Ethnic Medicine and TCM (Ministry of Education), Guizhou Provincial Key Laboratory of Pharmaceutics, School of Pharmacy, Guizhou Medical University, Guiyang, 550004 China

## Abstract

**Introduction:**

Maintaining protein homeostasis is vital for multiple myeloma (MM) cell survival. Indirubin- 3-monoxime (I3MO), a potential MM therapeutic, inhibits proteasome activity, while histone deacetylase 6 (HDAC6) regulates autophagy. We developed I3MV- 8b, an I3MO derivative, integrating an HDAC6 inhibitor moiety to enhance dual inhibition of proteasome and autophagy pathways.

**Methods:**

The anti-MM effects of I3MV- 8b were tested in vitro and in vivo. To identify downstream targets, RNA-seq and dual-luciferase reporter assays were performed. Additionally, ChIP-seq and IP-MS techniques were employed to elucidate the underlying molecular mechanism.

**Results:**

I3MV- 8b significantly suppressed MM cell proliferation and induced apoptosis. Combined with proteasome inhibitors, I3MV- 8b enhanced cytotoxicity by concurrently inhibiting proteasome and autophagy pathways. It reduced TRIM28 transcription, correlating with lower expression of proteasome subunits and autophagy-related genes. ChIP-seq revealed that TRIM28 binds to proteasome gene promoters, and its knockdown decreased proteasome subunit expression and activity. TRIM28 knockdown also impaired autophagosome formation. IP-MS and Co-IP assays showed TRIM28 interacted with 14–3 - 3ζ, a negative regulator of autophagy, promoting its ubiquitination and degradation. This interaction reduced autophagy regulation, further sensitizing cells to treatment.

**Conclusions:**

I3MV- 8b offers a novel dual inhibition strategy targeting proteasome and autophagy, presenting a promising therapeutic option for MM.

**Graphical Abstract:**

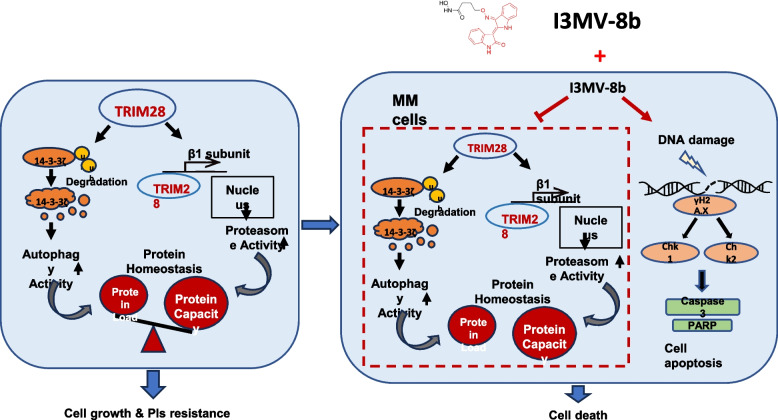

**Supplementary Information:**

The online version contains supplementary material available at 10.1186/s40364-025-00773-3.

## Introduction

Multiple myeloma (MM), the second most common hematological cancer, is characterized by the infiltration of malignant plasma cells into the bone marrow [1]. Despite the availability of effective therapeutic protocols for MM patients, such as proteasome inhibitors (PIs) known for their significant efficacy against this disease, inherent and acquired resistance to PIs remains a formidable challenge [2]. In the clinical setting, there is an urgent need to comprehend the genetic composition of these drug-resistant myeloma cells. This understanding is crucial to facilitate the development of innovative agents that can effectively treat patients with MM.

Paraproteins are abundantly produced by myeloma cells, establishing a strong reliance on cellular mechanisms such as the ubiquitin–proteasome system (UPS), autophagy, and the unfolded protein response (UPR) to maintain cellular homeostasis [3]. As the catalytic core of the UPS, proteasome plays a crucial role in intracellular protein degradation [4]. Pharmacologically inhibiting the proteasome has been shown to induce cell death in cancer cells. Proteasome inhibitors (PIs) have effectively been utilized to achieve therapeutic benefits in MM treatment, resulting in durable responses that significantly improve patient survival [5–7]. While PIs are known to stimulate polyubiquitinated proteins accumulation, they also trigger aggresomes formation and activate autophagy. These processes aim to enhance protein clearance, support tumor cell survival, and contribute to PIs resistance development [8–10]. Research studies have provided evidence that inhibiting autophagy can increase PIs sensitivity and induce cell death in MM [11–15]. Combining inhibition of both the proteasome pathway and autophagy pathway represents a promising therapeutic strategy for MM.

Our previous study has demonstrated the efficacy of indirubin- 3-monoxime (I3MO) as a proteasome inhibitor against MM cell proteasome activity by targeting proteasome activators PSME3 and PSME4 [16]. Recent studies have shown promising results inhibiting the aggresome-autophagy pathway and enhancing PIs-induced multiple myeloma cell death through histone deacetylase 6 (HDAC6) inhibitors [17–21]. Therefore, our aim is to investigate the potential synergistic anti-myeloma effects of I3MO and HDAC6 inhibitors by synthesizing a novel compound termed I3MV- 8b (hereafter referred to as 8b) that incorporates the moiety of an HDAC6 inhibitor into the structure of I3MO [22].

TRIM28, a member of the TRIM protein family, is part of the largest subfamilies of E3 ligases [23]. Previous studies have demonstrated that TRIM28 functions as an E3 ubiquitin ligase, targeting phosphorylated RB protein, p53, and AMPK for degradation through proteasome-dependent pathways [24–27]. As a result, TRIM28 plays a crucial role in regulating essential biological processes such as cell proliferation, and genome stability in various cancers [26, 28]. Most importantly, research showed that TRIM28 is a critical autophagy modulator. Furthermore, TRIM28 acts as a significant transcriptional regulator and translocate into the nucleus to activate the transcription of several key proteasome subunits, which counteracts the inhibitory effects of bortezomib (BTZ) on the proteasome in hepatocellular carcinoma [23, 29, 30]. Although there is evidence linking TRIM28 to protein homeostasis regulatory pathways, including proteasome and autophagy, its function and potential as a therapeutic target in multiple myeloma have not been previously clarified.

In this study, we synthesized a novel derivative 8b by conjugating the moiety of an HDAC6 inhibitor into the I3MO structure. We comprehensively evaluated the efficacy and underlying mechanisms of 8b in inducing cytotoxicity and synergistic effects with PIs against MM cells both in vitro and in vivo. Furthermore, our research identified TRIM28 as a potential target of 8b, providing novel insights into the role and mechanism of TRIM28 as a protein homeostasis regulator in MM pathogenesis. These findings highlight the potential therapeutic significance of targeting TRIM28 for MM treatment.

## Material and methods

### Cell lines and primary patient samples

The human MM cell lines used in this study were ARP1 (CVCL_D523), U266 (CVCL_0566), RPMI8226 (CVCL_0014), KMS11 (CVCL_2989). These cell lines were cultured in RPMI- 1640 medium (Gibco, C11875500BT, USA) supplemented with 10% fetal bovine serum (Gibco, 10,100,147, AUS), 100 units/mL penicillin, and 100 mg/mL streptomycin (Gibco, 15140122). STR profiling was performed to authenticate the cell lines, and they were maintained under mycoplasma-free conditions at 37 °C with 5% CO_2_. Primary MM cells were obtained from patients with newly diagnosed multiple myeloma (NDMM) and relapsed/refractory multiple myeloma (RRMM) who visited our hospital. The study protocol was conducted in accordance with the principles outlined in the Declaration of Helsinki and was approved by the local institutional Ethics Committees (reference number: KT2020010-EC2). Informed consent was obtained from all participants who agreed to participate in the study. The collected data were anonymized for analysis purposes.

### Reagents

Compound 8b was synthesized as a stock solution of 100 mM in dimethyl-sulfoxide (DMSO) obtained from ChenPartner chemicals (Shanghai, China). The stock solution was stored at − 20 °C and further diluted to the desired concentrations in each well plate containing the cell suspension. Indirubin- 30-monoxime (I3MO, HY- 19807, CAS Number: 160807–49 - 8), Tubacin (Tub, HY- 13428, CAS Number: 537049–40 - 4), MG132 (HY- 13259, CAS Number: 133407–82 - 6), and Chloroquine (CQ, HY- 17589 A, CAS Number: 54–05–7) were obtained from MCE. Bortezomib (PS- 341, Velcade) was purchased from Selleck Chemicals.

### RNA sequencing and GEO dataset analysis

A panel of MM cell lines, ARP1, U266, and RPMI 8226, were treated with 8b for 24 h, and RNA sequencing was performed. MM cell lines (ARP1, RPMI 8226, and U266) were harvested after TRIM28 knocking down and subjected to RNA sequencing as previous described [16]. Biological enrichment analysis was conducted using GO, KEGG, and Reactome gene sets with the cluster Profiler 4 tool [31]. Pearson correlation analysis was used to investigate the correlation between TRIM28 and proteasome subunit genes as well as autophagy-related genes. The clinical significance of TRIM28 was investigated using GEO datasets GSE5900, GSE2658, and the TCGA data of MMRF-CoMMpass. Correlation analysis was performed using GEO datasets GSE31161, GSE24080, and GSE2658.

### Proteasome activity assay

Proteasome-Glo™ Chymotrypsin-Like, Caspase-Like, and Trypsin-Like Cell-Based Assays (G8660, Promega Corporation, Madison, USA) were used to conduct proteasome activity assays following the manufacturer’s protocol.

### Dual‑luciferase reporter assay

Luciferase (pGL3-TRIM28-Luc, YouBio), and Renilla (pRL-TK, YouBio) vectors were transfected into HEK293 T and KMS11 cells using X-tremeGENE HP (Roche). After 24 h, cells were treated with 8b and then harvested for a luciferase reporter assay performed with the Dual-Luciferase Reporter Assay System (Promega), following the manufacturer’s guidelines. Readings were taken using a microplate reader. All samples were tested in triplicate.

### Tumor xenograft model

**Fig. 1:**
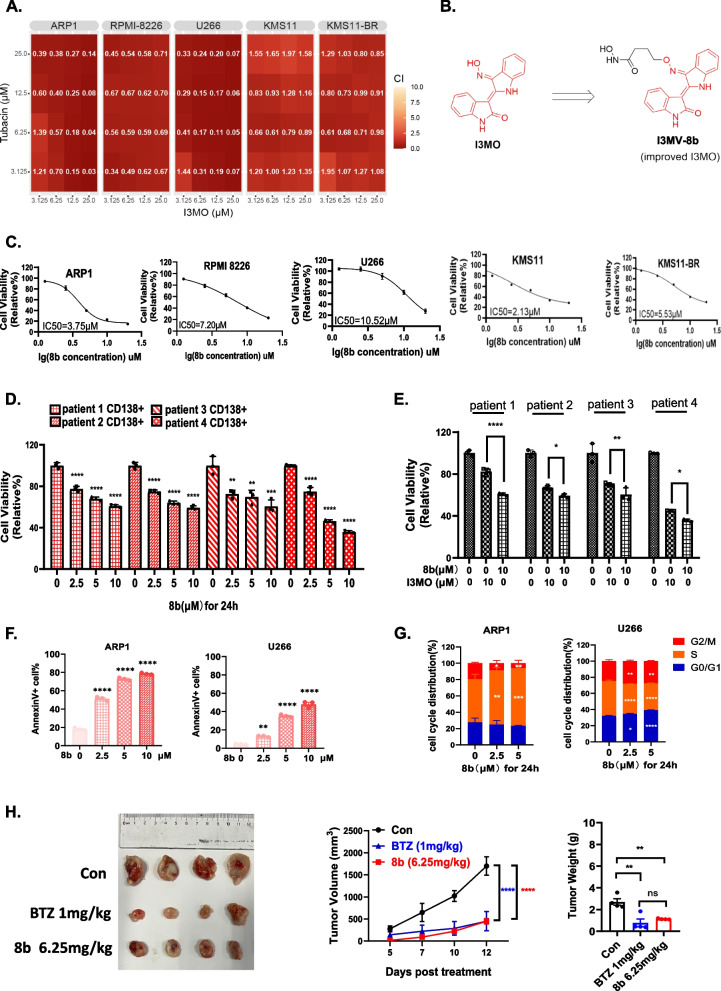
I3MO combined with HDAC6 inhibitor enhance the anti-MM cytotoxicity. **A** Heatmap showing the combination index (CI) values of I3MO and Tubacin (Tub) in MM cell lines. The cells were treated with various combinations of I3MO and Tub at different doses, and the CI values were calculated using the Chou-Talalay method. The heatmap color scale represents the CI values (indicated on the graph), with red indicating synergistic effect (CI < 1.0). **B** Chemical structure of I3MV- 8b, which is synthesized by incorporating the moiety of an HDAC6 inhibitor into the I3MO structure (Red) through a linker. **C** The dose–response curves of MM cell lines ARP1, RPMI 8226, U266, KMS11 and KMS11-BR to 8b were plotted based on the viability assay data. The cells were treated with increasing concentrations of 8b for 24 h, and cell viability was measured using CCK8 assay. The IC_50_ values for each cell line are indicated on the graph. **D** Bar plots showing different cytotoxic effect of 8b on primary CD138^+^ cells of bone marrow aspirates from 4 MM patients. The viability of primary cells as measured using CCK8 assays after treatment with different concentrations of 8b (0, 2.5 μM, 5 μM and 10 μM) for 72 h. **E** Bar plots showing different cytotoxic effect of 8b and I3MO on primary CD138^+^ cells of bone marrow aspirates from 4 MM patients. The viability of primary cells as measured using CCK8 assays after treatment with 10 μM 8b or I3MO for 72 h. **F** Bar plots showing the percentage of apoptotic cells in MM cell lines ARP1 and U266 after treatment of DMSO control, 2.5 μM, 5 μM, 10 μM 8b for 24 h. each group as determined by flow cytometry analysis. **G** Bar plots showing cell cycle distribution in MM cell lines ARP1 and U266 by flow cytometry after treatment of DMSO control, 2.5 μM and 5 μM 8b for 24 h. The results are presented as percentage of cells in each phase of the cell cycle (G0/G1, S, and G2/M) calculated by Flow jo software. **H** The tumors were harvested at the end of the study, photographed and weighed. Representative tumor pictures of xenografts from mice treated with DMSO control, 6.25 mg/kg 8b, or 1 mg/kg Bortezomib. The treatment groups are labeled on the picture. The ruler on the picture indicates the size of the tumor in millimeters. Line chart showing the changes in tumor volume over time in mice bearing MM xenografts under the treatment of DMSO control, 6.25 mg/kg 8b, or 1 mg/kg BTZ. * *P* < 0.05, ** *P* < 0.01, *** *P* < 0.001, **** *P* < 0.0001 (two-way ANOVA test). Bar plot displaying mean ± SEM tumor weight of xenografts from each group. * *P* < 0.05, ** *P* < 0.01, *** *P* < 0.001, **** *P* < 0.0001 (one-way ANOVA followed test)

A total of 1 × 10^6^ ARP1 MM cells were injected subcutaneously into the left flanks of 6 to 8 weeks old female NOD/SCID mice. After 10 days, the mice were randomly assigned to groups for treatment with various agents. Tumor volume was measured every 3 or 4 days as the tumor burden according to the formula tumor volume = 1/2 × length x width^2^ (Figs. 1H, 4B and 6G).

NOD-Rag1null mice 6—8 weeks old were injected with 1 × 10^6^ ARP1 expressing luciferase via tail vein injection. After 10 days, mice were randomly assigned to groups for treatment with various agents. Tumor burden was assessed at the end point of the experiment by imaging each mouse using Carestream IN-Vivo MS FX Pro with constant exposure time. The pixel intensity in selected areas around each mouse was quantified (Fig. 4C).

### Plasmids and transfection

The doxycycline-inducible vector PLKO-Tet-on was used to insert shRNA sequences targeting TRIM28. TRIM28 was then subcloned into the pCDH vector and tagged with 3 × FLAG. Lentivirus was produced in HEK293 T cells (RRID: CVCL_HA71) involved pMD2.G and psPAX2 helper vectors (Addgene), along with X-tremeGENE HP(Roche). After 72 h, the HEK293 T cells were harvested and concentrated tenfold using the Lenti-X Concentrator from Takara Bio USA, Inc. Subsequently, 0.5 × 10^6^ cells were plated in a 24-well plate and exposure to lentivirus along with polybrene (8 ug/ml, Santa). Stable cells were selected following the manufacturer's protocol for assessing target protein expression through western blot analysis.

### ChIP-qPCR

The binding of TRIM28 to PSMB1 in ARP1 and U266 was quantified by ChIP-qPCR following the manufacturer’s guidelines provided with the Thermo Scientific Pierce Magnetic ChIP Kit (#26,157) The cells were crosslinked by adding formaldehyde at a final concentration of 3.7% and incubating for 15 min at room temperature to bind the proteins to DNA. Crosslinking was stopped by adding 125 mM Glycine, followed by three washes to remove excess reagents. SDS lysis buffer was used to lyse the cells, which were further disrupted using an ultrasonic cell disruptor. For the ChIP assay, chromatin samples (5 μg) from ARP1 and U266 myeloma cell lines were immunoprecipitation using antibodies specific to TRIM28 (Abcam Cat# ab10483, RRID: AB_297222). Enrichment of DNA fragments containing putative TRIM28 binding sites in the gene promoter region was determined by qPCR using a Real-Time PCR Detection System (Bio-Rad). The primers designed for PSMB1 in the ChIP-qPCR assay are as follows: Primer1: forward, GGGAAAATCAAGGCGTGCTG; reverse, GGCTGTCGGGAGATTGACAG. Primer2: forward: AGATCAGCTAGCGTTCTTGC; reverse, AAAGCAACTAGCGGCGTTAC.

### Co-IP

A total of 2 × 10^7^ ARP1, RPMI 8226, and U266 cells were lysed in NP- 40 lysis buffer containing protease inhibitors. Cell extracts were precleared with protein A/G beads before incubating overnight at 4 °C with anti-TRIM28 (Abcam Cat# ab10483, RRID: AB_297222) or anti- 14–3 - 3ζ (14,881–1-AP, RRID: AB_2218248). Protein A/G beads were added and further incubated for 4 h. The beads underwent five washing buffer, following by the addition of 50 µl of 1 × SDS loading buffer. Samples were boiled at 95–100 °C for 5 min, subsequently analyzed by western blot.

### Immuno-staining and fluorescent microscopy

The tumor cells on slides were fixed and permeabilized using Cytofix/Cytoperm reagents (BD). Then, a blocking buffer containing 0.1% saponin and 5% donkey serum was applied to the cells. After blocking step, the cells were stained with a TRIM28 antibody (Abcam Cat# ab10483, RRID: AB_297222) for 1 h at room temperature. Following thorough washing, the cells on the slides were incubated with Alexa Fluor 647-conjugated secondary antibodies (diluted at a ratio of 1:100) in a dark environment. Finally, the slides were washed three times, mounted using ProLong Gold anti-fade reagent with DAPI (Invitrogen), and observed under a fluorescent microscope.

### ELISA assay

The density of 5 × 10^5^ MM cells/mL was incubated in complete medium for 24 h, and the levels of human λ or κ light chain were assessed in the cell culture supernatant using the manufacturer's protocol (#E88 - 115 and #E88 - 116; Bethel Laboratories).

### Statistical analysis

Data presentation involved mean ± SD or mean ± SEM, as specified in the figure legends. Statistical evaluations were conducted using GraphPad Prism software (version 8.01, GraphPad Software Inc.). A two-tailed Student’s t-test was employed for comparisons between two groups, while an ANOVA was applied for cases involving more than two groups. Survival analyses were carried out using Kaplan–Meier methods. Differentially expressed genes (DEGs) post 8b treatment or following TRIM28 knockdown were identified using the EdgeR package in R, with a log (Fold Change) threshold ≥ 0.6. Statistical significance was determined at P values of < 0.05 (*), < 0.01 (**), < 0.001 (***), and < 0.0001 (****).

## Results

### The combination of I3MO and HDAC6 inhibitor enhances the cytotoxicity effect against MM

It is critical for myeloma cells to maintain proteostasis to ensure their survival. Our previous study demonstrated that the I3MO, a novel proteasome inhibitor, effectively inhibits the growth of MM cells [16]. HDAC6 is involved in the autophagy degradation pathway for misfolded proteins. Dual inhibition of histone deacetylase and proteasome is an attractive strategy for myeloma therapy. In this study, we evaluated the anti-myeloma ability of I3MO in synergy with the HDAC6 inhibitor Tubacin (Tub), which showed a potent anti-myeloma effect (Fig. 1A). Importantly, the combination of Tub and I3MO exhibited synergistic cytotoxicity even in the BTZ-resistant KMS11-BR cells (Fig. 1A). To further improve the potency of I3MO, we synthesized a compound called I3MV- 8b (8b) by conjugating the moiety of an HDAC6 inhibitor into the structure of I3MO (Fig. 1B) [22]. The cytotoxicity of 8b was observed in both MM cell lines and primary samples from MM patients, with IC_50_ values at 48 h being 3.75 μM, 7.2 μM, and 10.52 μM in ARP1, RPMI 8226, and U266 cells, respectively (Fig. 1C). Furthermore, 8b displayed similar cell cytotoxic effects on both BTZ-resistance and sensitive cell lines including KMS11-BR and KMS11; with IC_50_ value being 5.53 μM and 2.13 μM, respectively, indicating its ability to overcome BTZ resistance (Fig. 1C). Our findings demonstrated that compound 8b effectively suppressed the growth of patient primary CD138^+^ MM cells (*n* = 4, *P* < 0.05,* t* test, Fig. 1D) in a dose-dependent manner. Notably, 8b exhibited stronger cytotoxicity than I3MO in MM patient samples (Fig. 1E). Importantly, no significant cytotoxicity was observed on normal peripheral blood mononuclear cells (PBMCs) upon treatment with 8b (Fig. S1A). This favorable specificity in targeting MM cells and the absence of cytotoxicity on normal cells highlight the potential therapeutic benefits of 8b. Flow cytometry analysis demonstrated that 8b treatment significantly induces apoptosis and cell cycle arrest in MM cells (Fig. 1F & G & Fig. S1B & S1C). Western blot analysis further confirms that 8b activates the caspase-dependent endogenous apoptosis pathway, as evidenced by increased levels of cleaved caspase 3 and PARP upon treatment with 8b. Additionally, DNA damage-related markers including γH2 AX, pCHK1, and pCHK2, were elevated in MM cells treated with 8b, suggesting that induction of DNA damage is also involved in the cytotoxicity effects of 8b treatment in MM cells (Fig. S1D).

To confirm the therapeutic cytotoxicity of compound 8b, a myeloma xenograft murine model was utilized. As shown in Fig. 1H, treatment with 8b significantly reduced tumor size compared to the PBS control group, with BTZ treatment as the positive control. Our findings also demonstrated that treatment with 8b did not cause significant body weight loss, indicating good tolerance and effective anti-MM effects of 8b (Fig. S1E). We routinely monitored body weight and white blood cell counts in Balb/c mice receiving 8b treatment three times a week to evaluate its potential toxicity. Results showed no significant hematological toxicity or weight loss associated with 8b treatment (Fig. S1F & G).

### Compound 8b efficiently suppresses the proteasome activity in MM

**Fig. 2:**
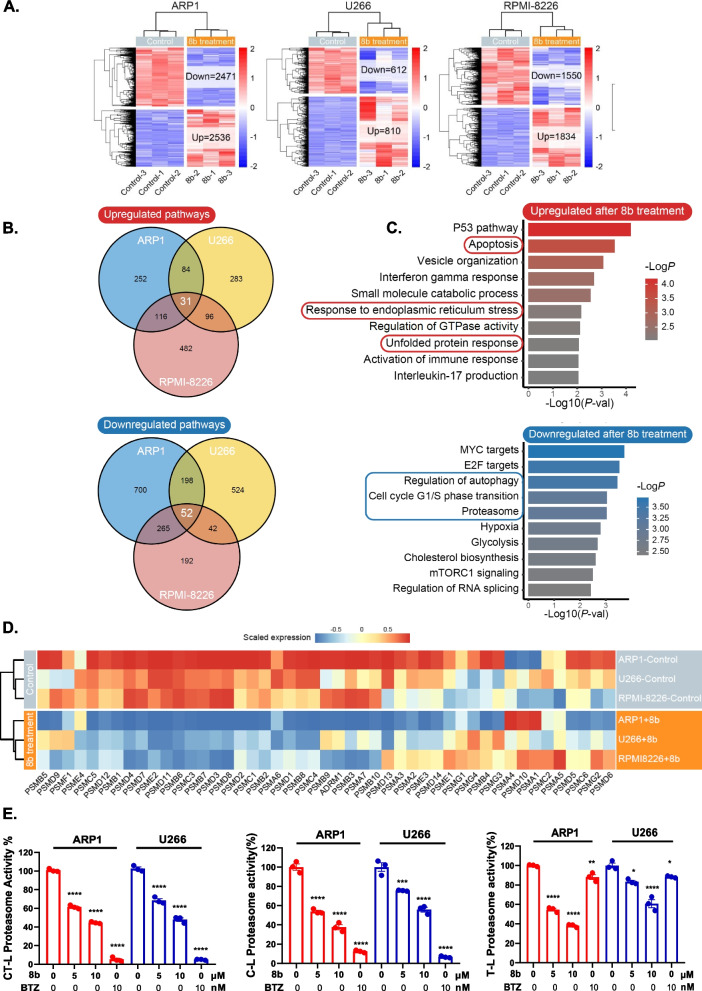
Compound 8b suppresses the proteasome activity in MM cells. **A** Heatmap illustrating the differential expression of genes in ARP1, RPMI 8226, and U266 cell lines after treatment with 8b or DMSO control for 24 h. **B** Venn plot illustrating the extent of overlap in the upregulated and downregulated pathways across cell lines following exposure to 8b treatment. **C** Bar plot displaying the Top10 pathways that are commonly upregulated and downregulated across various cell lines following exposure to 8b treatment. **D** Heatmap illustrating the differential expression of proteasome subunits in ARP1, RPMI 8226, and U266 after treatment with 5 μM 8b or DMSO control. **E** Bar plot showing chymotrypsin-like (CT-L), caspase-like (C-L), and trypsin-like (T-L) proteasome activity in ARP1 and U266 cell lines after treatment with DMSO control, 8b (5 μM, and 10 μM) for 24 h, or 10 nM Bortezomib for 12 h. The proteasome activities were measured using fluorogenic peptide substrates, and each bar represents the mean ± SEM of triplicates. * *P* < 0.05, ** *P* < 0.01, *** *P* < 0.001 (one-way ANOVA test)

To investigate the molecular mechanism underlying the anti-MM ability of compound 8b, we initially conducted RNA-seq analysis. Heatmaps were generated to visually represent the differential gene expression (DEGs) in MM cells treated with 8b across multiple MM cell lines (FDR < 0.05, |log_2_FoldChange|> = 0.6, edgeR). In ARP1 cells, there were 5,007 DEGs (2,536 upregulated and 2,471 downregulated); in RPMI 8226 cells, there were 3,384 DEGs (1,834 upregulated and 1,550 downregulated), and in U266 cells, a total of 1,422 DEGs (810 upregulated and 612 downregulated), all following treatment with wither a concentration of 5 μM or 10 μM for 24 h (Fig. 2A). Subsequently, pathway enrichment analysis using GO, KEGG, and Reactome gene sets was performed on the DEGs to identify biological pathways that were commonly upregulated or downregulated among all four examined MM cell lines. A total of 31 biological pathways showed common upregulation (*P* < 0.05), while 52 pathways exhibited common downregulation (*P* < 0.05, Fig. 2B). Among the upregulated pathways, the P53 pathway and apoptosis pathway emerged as particularly significant. Conversely, the downregulated pathways included the MYC pathway, E2 F pathway, and cell cycle transition pathway. Intriguingly, the response to endoplasmic reticulum (ER) stress and the unfolded protein response were upregulated, indicating impairment in the protein degradation pathway. Consistently, protein degradation-related pathways, including autophagy and the proteasome, were significantly downregulated (Fig. 2C). Importantly, upon treatment with 8b, multiple proteasome subunits were consistently downregulated in all three MMCLs (Fig. 2D), which aligns with previous RNA-seq data of MM cell lines treated with I3MO. Subsequently, we selected the ARP1 and U266 cell lines, which represent cells with sensitive and less sensitive responses to 8b, respectively, for downstream pathway validation. Decreased proteasome activities, including chymotrypsin-like (CT-L) and caspase-like (C-L) activities, were observed in both ARP1 and U266 cell lines following treatment with 8b. BTZ treatment was used as a positive control (Fig. 2E). Interestingly, trypsin-like (T-L) proteasome activity was also downregulated after treatment with 8b but not affected by BTZ. These findings collectively support the hypothesis that 8b acts as a novel proteasome inhibitor by downregulating proteasome subunits and inhibiting proteasome activity in a manner distinct from that of BTZ.

### Compound 8b suppresses autophagy by disrupting aggresome and autophagosome formation

**Fig. 3:**
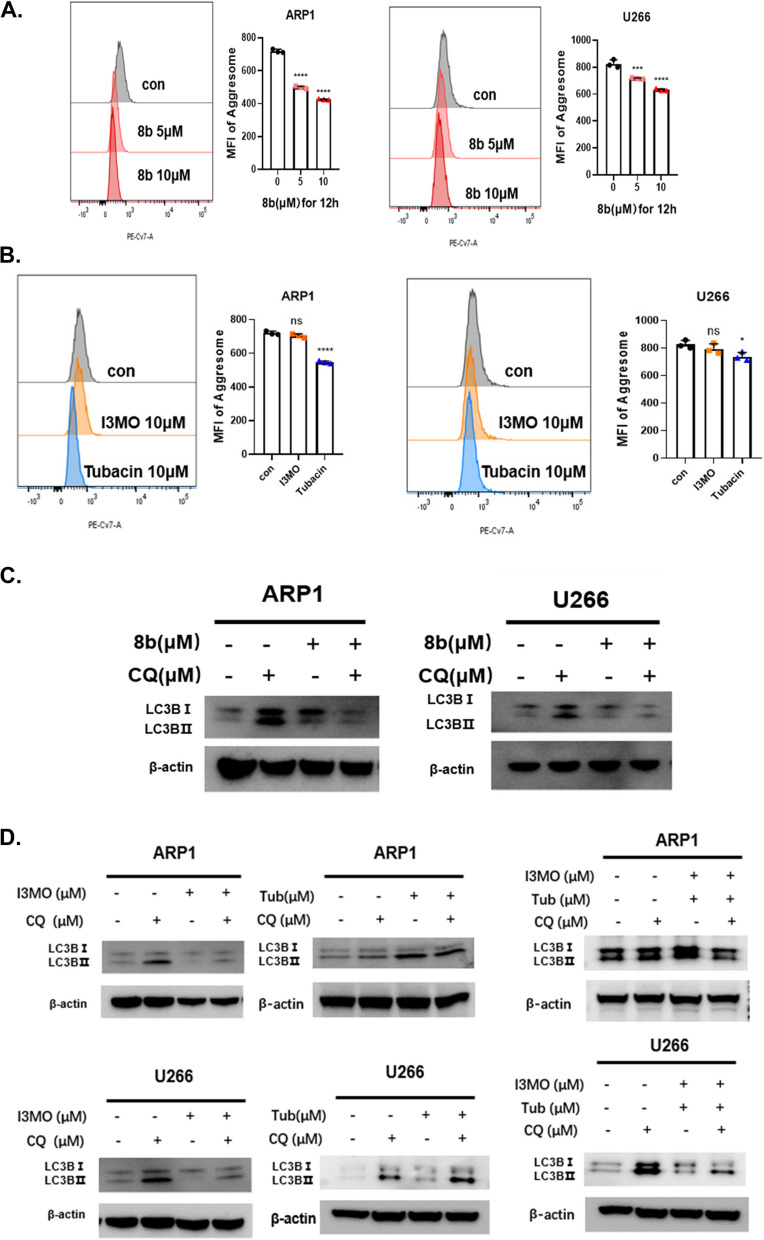
Compound 8b suppresses autophagy by disrupting aggresome and autophagosome formation. **A** (Left panel) Flow cytometry histogram showing the level of aggresome in MM cell lines ARP1 and U266 after treatment with DMSO control and 8b (5 μM and 10 μM) for 6 h. Aggresomes were detected using a molecular rotor dye from the ProteoStat Aggresome Detection Kit. (Right panel) Bar plots showing the mean fluorescence intensity (MFI) in corresponding cell lines and conditions. Each bar represents the mean MFI ± SEM of triplicate measurements for each group. * *P* < 0.05, ** *P* < 0.01, *** *P* < 0.001 (one-way ANOVA test). **B** Flow cytometry histogram showing the level of aggresome in MM cell lines ARP1 and U266 after treatment with DMSO control and I3MO (10 μM) and Tubacin (10 μM) for 6 h. Bar plots showing the mean fluorescence intensity (MFI) in corresponding cell lines and conditions. The data are triplets and shown as mean ± SEM. * *P* < 0.05, ** *P* < 0.01, *** *P* < 0.001, **** *P* < 0.0001 (one-way ANOVA test). **C** Western blots showing the protein levels of LC3BI, LC3BII, and β-actin in MM cell lines ARP1 and U266 after -treatment with DMSO control or 5 μM 8b with or without Chloroquine (CQ) for 24 h. The CQ was used to block the lysosomal degradation of autophagosomes. β-actin was used as a loading control. **D** Western blots showing the protein levels of LC3BI, LC3BII, and β-actin in MM cell lines ARP1 and U266 after treatment with DMSO control, 10 μM I3MO, 10 μM Tub, or co-treatment with 10 μM I3MO and 10 μM Tub with or without Chloroquine (CQ) for 24 h

The previous report indicated that the aggresome pathway is activated when misfolded proteins cannot be cleared by the proteasome [8–10]. HDAC6 can transport ubiquitinated pathological aggregates to the microtubule-organizing center (MTOC) for aggresome formation and autophagosomal degradation [32, 33]. As a dual inhibitor of HDAC6 and the proteasome, we investigated how 8b affects aggresome formation and autophagy activation. Flow cytometry analysis revealed that 8b significantly suppressed the formation of aggresome in a dose-dependent manner, which is detected by a novel red-fluorescent dye specific to aggresome (Fig. 3A). Among the two parent compounds of 8b, only Tub significantly inhibited aggresome formation through its HDAC6 inhibition effect, while I3MO had limited impact on aggresome formation. This finding suggests that the inhibitory effect of 8b on aggresomes relies on its ability to inhibit HDAC6 (Fig. 3B).

After aggresome formation, it undergoes further degradation through downstream autophagy pathways. Our RNA-seq data showed that 8b treatment downregulates autophagy, so we investigated its impact on autophagy process. Since LC3BII is degraded by autophagy, different steady-state levels of LC3BII can indicate either activation or inhibition of autophagy. To distinguish between these possibilities, cells were treated with chloroquine (CQ) to block lysosomal-dependent protein degradation in autophagosomes. In the presence of CQ, both ARP1 and U266 cell lines exhibited decreased LC3BII levels, indicating that 8b treatment suppresses autophagosomes formation (Fig. 3C). Our results also show that I3MO treatment hinders autophagosome formation while Tub enhances it. The combination of I3MO and Tub leads a resultant suppression of autophagosome formation similar to the effects of 8b treatment (Fig. 3D).

### Compound 8b synergistically enhances cytotoxicity of proteasome inhibitors

**Fig. 4:**
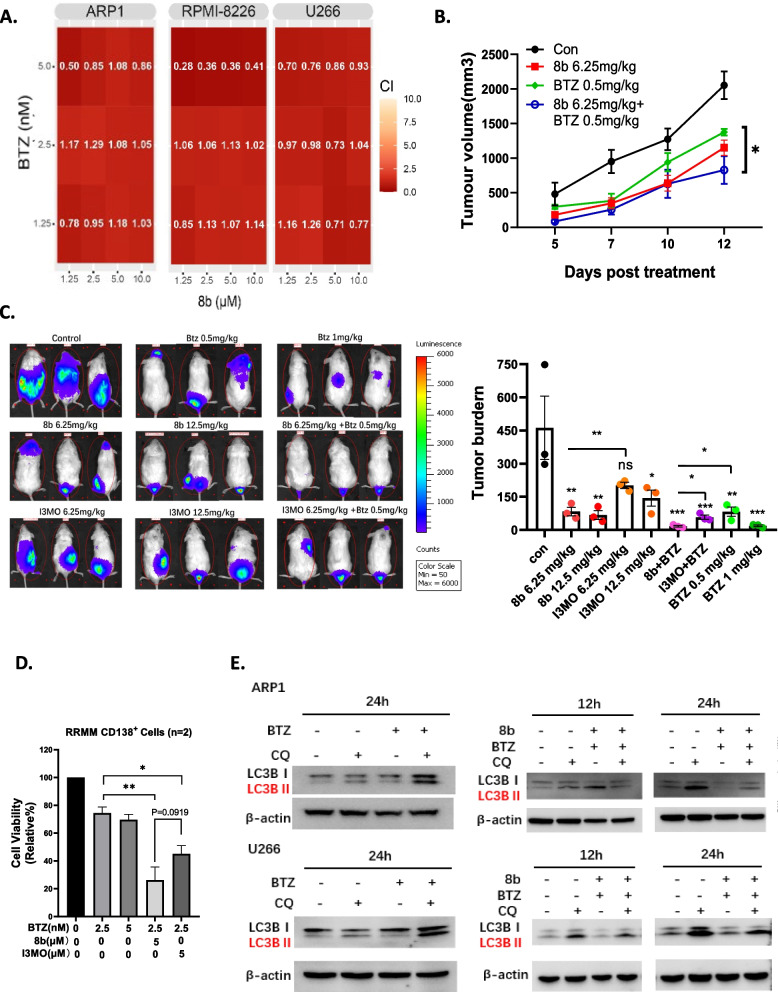
Compound 8b synergistically enhances cytotoxicity of proteasome inhibitors. **A** Heatmap showing the combination index (CI) values of 8b and Bortezomib in MM cell lines ARP1, RPMI8226, and U266. The CI values were calculated using the Chou-Talalay method. The heatmap color scale represents the CI values (indicated on the graph), with red indicating synergistic effect (CI < 1.0). **B** Line chart showing the changes in tumor volume over time in mice bearing MM xenografts under the treatment of DMSO control, 6.25 mg/kg 8b, 0.5 mg/kg BTZ, or combination of 6.25 mg/kg 8b and 0.5 mg/kg BTZ. Tumor volume was measured every three days starting from the treatment. The data points represent the mean ± SEM of 4 mice per group. * *P* < 0.05, ** *P* < 0.01, *** *P* < 0.001, **** *P* < 0.0001 (two-way ANOVA test). **C** (Left) In vivo imaging showing the tumor growth in the different groups of mice after treatments at end point of the experiment. (Right) Quantification of tumor burden was determined and Dunnett’s method was used to calculate the multiplicity-adjusted P values for each treatment and control group after 3 weeks of treatment. * *P* < 0.05, ** *P* < 0.01, *** *P* < 0.001, **** *P* < 0.0001 (one-way ANOVA test). **D** Bar plots showing different cytotoxic effect of BTZ, combination of 8b and BTZ or combination of I3MO and BTZ on primary CD138^+^ cells of bone marrow aspirates from 2 MM patients. The viability of primary cells as measured using CCK8 assays after treatment with different drugs for 72 h. The results are presented as percentage of cell viability compared to untreated cells. The data are triplets and shown as mean ± SEM. * *P* < 0.05, ** *P* < 0.01, *** *P* < 0.001, **** *P* < 0.0001 (one-way ANOVA test). **E** Western blots showing the protein levels of LC3BI, LC3BII, and β-actin in MM cell lines ARP1 and U266 after treatment with DMSO control, 5 nm BTZ, 5 μM 8b, or co-treatment with 8b and BTZ with or without Chloroquine (CQ) for 24 h

To investigate the potential synergistic effects of combining 8b with BTZ, combination studies were performed, and the combination index (CI) values were calculated using CompuSyn software. The data demonstrated that 8b significantly enhanced the sensitivity of MM cells to BTZ-induced apoptosis, indicating in vitro synergistic effects between 8b and BTZ. This was supported by CI values below 1.0 (Fig. 4A). Furthermore, a xenograft myeloma murine model demonstrated that the combination treatment group, consisting of 6.25 mg/kg 8b and 0.5 mg/kg BTZ, synergistically suppressed tumor burden compared to treatment with either compound alone (Fig. 4B). Additionally, an ARP1-Luc disseminated (IV) myeloma mode was utilized to evaluate tumor load through whole-body bioluminescence signal measurement. A combined treatment of BTZ with 8b caused an additive reduction in tumor burden compared to the monotherapy. Notably, both the efficacy of 8b as a monotherapy and its combination effects with BTZ were superior to those observed with I3MO compounds (Fig. 4C). Furthermore, the synergistic effects of BTZ and 8b were investigated in CD138^+^ primary MM patient cells. Consistently, the combination therapy induced a synergistically enhanced cytotoxicity compared with single-agent administration of 8b and the combined effects of 8b with BTZ were superior to those of I3MO with BTZ (Fig. 4D). Recent studies have suggested that BTZ-induced autophagy is one of the mechanisms underlying acquired drug resistance in MM cells [34, 35]. Therefore, we postulated that the synergistically enhancement of cytotoxicity by PIs would be attributed to the inhibition of autophagy upon treatment with compound 8b. Intriguingly, administration of 8b effectively suppressed BTZ-induced autophagy as evidenced by reduced expression levels of LC3BII in MM cell lines (Fig. 4E). These findings imply that 8b potentiates PIs’ cytotoxicity against MM through suppression of induced autophagy triggered by PIs.

### TRIM28 is a crucial downstream target of compound 8b

**Fig. 5:**
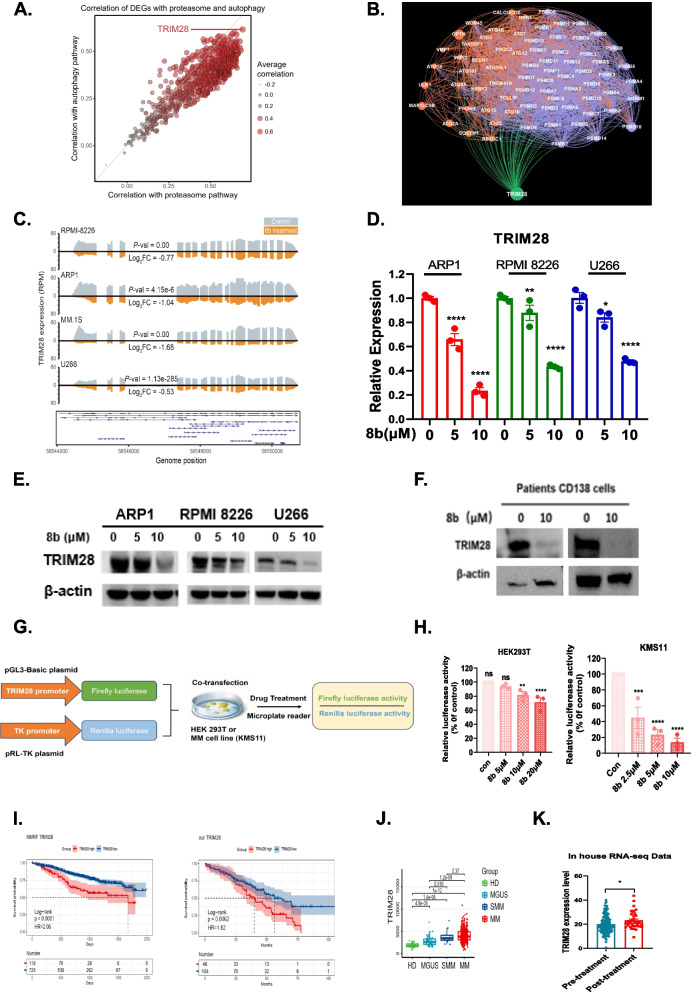
TRIM28 is a key downstream target of 8b. **A** The dot plot shows the average correlation between 744 genes downregulated in at least three cell lines and genes in the proteasome and autophagy pathways, with the size and color of the dots representing the Pearson correlation coefficient. **B** The network graph displays the correlation between TRIM28 and genes of the proteasome (purple) and autophagy (orange) pathways, where only edges with a correlation coefficient > 0.4 are retained. The size of the dots represents the degree of gene expression reduction after 8b treatment. **C** Peak plot depicting TRIM28 expression in three multiple myeloma cell lines (ARP1, RPMI8226, and U266) after treatment with 8b at a concentration of 5 μM or 10 μM, as observed in RNA sequencing results. **D** RT- qPCR depicting TRIM28 mRNA level after treatment with DMSO control or 8b (5 μM and 10 μM) for 24 h in ARP1, RPMI8226, and U266 cell lines. **E** Western blots showing protein level of TRIM28 after treatment with DMSO control or 8b (5 μM and 10 μM) for 24 h in ARP1, RPMI8226, and U266 cell lines. **F** Western blots showing protein level of TRIM28 after treatment with DMSO control or 8b (10 μM) for 24 h in primary CD138^+^ cells of bone marrow aspirates from MM patient. **G** Schematic graph of dual luciferase reporter assay. **H** Bar plots showing promoter activity of TRIM28 in the presence of 8b following 24 h treatment assessed by luciferase reporter assays in HEK293 T cells and KMS11 cells transfected with the control vector and the target sequence vector containing human TRIM28 promoter (− 2000/− 1) plasmids. The promoter activity of TRIM28 in the presence of 8b (5, 10, 20 μM) for 24 h in HEK293 T cells, and in the presence of 8b (2.5, 5, 10 μM), for 12 h in KMS11 cells were detected by luciferase reporter assays. **I** OS rates of myeloma patients with high or low TRIM28 expression were evaluated by Kaplan–Meier analysis in MMRF-CoMMpass clinical trial and our in-house RNA-seq dataset. **J** The expression of TRIM28 was compared in plasma cells from healthy donors (HD, *n* = 22), individuals with MGUS (*n* = 44), individuals with SMM (*n* = 12) and newly diagnosed MM patients (*n* = 414) from GSE5900 and GSE4581. **K** The expression of TRIM28 was compared in plasma cells from pre-treatment patients and post-treatment patients from our in-house RNA seq data. The data are triplets and shown as mean ± SEM. * *P* < 0.05, ** *P* < 0.01, *** *P* < 0.001 **** *P* < 0.0001 (one way ANOVA test)

To investigate the target of 8b treatment in dual inhibition of proteasome and autophagy, we further analyzed the RNA-seq data in MM cells treated with and without 8b. Firstly, correlation analyses were conducted between 744 genes that were downregulated in at least three cell lines treated with 8b based on RNA-seq data and genes associated with the proteasome pathway and autophagy pathway. Among these genes, TRIM28 exhibited the strongest correlation with genes from both pathways (Fig. 5A). Importantly, Spearman correlation analysis revealed a positive association between TRIM28 levels and the expression of various proteasome subunits, as well as multiple autophagy-related genes, in the CoMMpass MM patient dataset (Fig. 5B). In various other publicly available GEO datasets of MM patients, TRIM28 also exhibits widespread correlations with genes from both pathways (Fig. S2 A). Subsequently, consistent downregulation of TRIM28 was observed in all three MMCLs treated with 8b based on the RNA-seq data (Fig. 5C). qRT-PCR and western blots confirmed that TRIM28 was downregulated at both mRNA and protein levels upon treatment with 8b in MMCLs (Fig. 5D & E). Western blots also demonstrated that 8b treatment led to downregulation of TRIM28 in MM patient samples (Fig. 5F). To elucidate the specific mechanism by which 8b regulates gene expression of TRIM28, we performed a dual-luciferase reporter assay using HEK293 T cells and KMS11 MM cells (Fig. 5G). The data revealed a significant reduction in TRIM28 promoter activity following treatment with 8b in both HEK293 T and KMS11 cell lines, as evidenced by a decreased Firefly luciferase to Renilla luciferase ratio (Fig. 5H). These findings indicate that treatment with 8b interferes with promoter activity, resulting in the downregulation of TRIM28 expression.

In the clinic, Kaplan–Meier survival analysis revealed that the MM patients with high level of TRIM28 exhibited a poor outcome compared to those with low levels in both MMRF-CoMMpass and in-house datasets (HR = 2.06, *p* < 0.0001; HR = 1.82, *p* = 0.0062) (Fig. 5I), which were in the context of treatment with bortezomib. This finding suggests a potential association between elevated TRIM28 expression and resistance to PIs. Furthermore, we observed a significant upregulation of TRIM28 expression in MM patient samples compared to healthy donors. As shown in Fig. 5J, there was a gradual increase in TRIM28 expression as the disease progressed from monoclonal gammopathy of undetermined significance (MGUS) and smoldering multiple myeloma (SMM) to active MM, indicating that high levels of TRIM28 may be associated with disease progression in MM patients. RNA-seq data from CD138^+^ MM cells revealed an even higher level of TRIM28 expression post-treatment, suggesting its potential involvement in mediating resistance to PIs treatment (Fig. 5K).

### TRIM28 acts as a transcription regulator of proteasome subunits expression

**Fig. 6:**
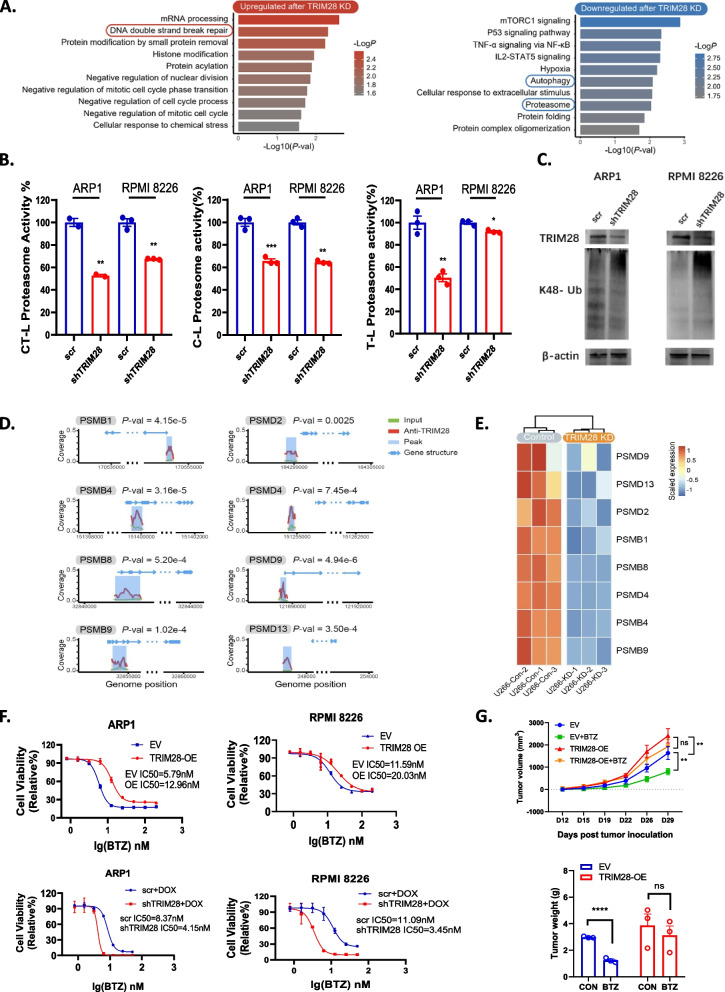
TRIM28 acts as a transcription regulator in regulation of proteasome subunits expression. **A** Bar plot displaying the Top10 pathways that are commonly upregulated and downregulated across distinct cell lines followingTRIM28 knockdown. **B** Bar plot showing chymotrypsin-like (CT-L), caspase-like (C-L), and trypsin-like (T-L) proteasome activity in TRIM28-KD MM cell lines. The proteasome activities were measured using fluorogenic peptide substrates, and each bar represents the mean ± SEM of triplicates. * *P* < 0.05, ** *P* < 0.01, *** *P* < 0.001, **** *P* < 0.0001 (one-way ANOVA test). **C** Western blots showing the levels of K48-polyubiquitination protein and TRIM28 levels in TRIM28-KD MM cell lines. **D** Peak plot showing the enrichment of TRIM28 in multiple proteasomes encoding genes promoter based on ChIP-seq data. **E** Heatmap displaying the downregulation of proteasome subunit-encoding genes in the U266 cell line following the knockdown of TRIM28, as observed in RNA-seq results. **F** The dose–response curves of MM cell lines to BTZ after overexpressing and knocking down TRIM28 were plotted based on the viability assay data. Cell viability was assessed by CCK8 after 24 h of treatment, and IC_50_ was calculated with GraphPad Prism 8. **G** Line chart showing the changes in tumor volume over time in mice bearing EV and TRIM28-OE MM xenografts under the treatment of DMSO control, and BTZ (0.5 mg/kg). Tumor volume was measured every three or four days starting from the 12 th day post tumor inoculation. The data points represent the mean ± SEM of 3 mice per group. * *P* < 0.05, ** *P* < 0.01, *** *P* < 0.001, **** *P* < 0.0001 (two-way ANOVA test followed by post-hoc Bonferroni test). Bar plot displaying mean ± SEM tumor weight of xenografts from each group. * *P* < 0.05, ** *P* < 0.01, *** *P* < 0.001, **** *P* < 0.0001 (one-way ANOVA test)

To investigate the function and mechanism of TRIM28 in MM, we employed knockdown (KD) and overexpression (OE) approaches in myeloma cell lines. Specifically, a doxycycline-inducible TRIM28-shRNA vector was transduced into ARP1, RPMI 8226, and U266 cell lines, while an ARP1 and RPMI 8226 cell line were transduced with 3xFlag-TRIM28 vector. Western blots analysis confirmed the levels of TRIM28 in the OE and KD cell lines (Fig. S2B & C). To investigate the biological function of TRIM28, RNA-seq was performed on MM cells after TRIM28 KD. Volcano maps were generated to visualize differentially expressed genes (DEGs) in MM cells following TRIM28 knockdown for various MM cell lines (FDR < 0.05, |log2 FoldChange|> = 0.6, edgeR). We observed a total of 2,116 DEGs (743 upregulated and 1,373 downregulated) in ARP1, 3,774 DEGs (2,344 upregulated and 1,430 downregulated) in U266, and 3,511 DEGs (1,548 upregulated and 1,963 downregulated) in RPMI 8226 after TRIM28 KD (Fig. S2D). Pathway enrichment analysis using GO, KEGG, and Reactome gene sets revealed commonly regulated pathways, including 21 upregulated pathways (*P* < 0.05), and 26 downregulated pathways (*P* < 0.05) among all three MMCLs (Fig. S2E). Notably, the proteasome pathway and autophagy pathway were both found to be downregulated following TRIM28 KD (Fig. 6A). These findings support the critical role of TRIM28 in regulating proteostasis through both proteasome and autophagy pathways.

Consistent with the treatment of 8b, a significant decrease in chymotrypsin-like (CT-L), caspase-like (C-L), and trypsin-like (T-L) proteasome activities was observed in ARP1 and RPMI 8226 cells upon TRIM28 KD, as revealed by the proteasome activity assay (Fig. 6B). Additionally, TRIM28-KD cells showed an accumulation of K48-polyubiquitinated proteins (Fig. 6C). Given that TRIM28 is a chromatin-associated protein functioning as a transcriptional regulator, we hypothesize that TRIM28 localizes in the nucleus and activates the transcription of genes encoding proteasome subunits. Immunofluorescence assay confirmed the nuclear localization of TRIM28 (Fig. S3A). ChIP-seq data demonstrated that TRIM28 could bind to promoter regions of multiple genes encoding proteasome subunits, including PSMB1, PSMB4, PSMB8, PSMB9, PSMD2, PSMD4, PSMD9 and PSMD13 (Fig. 6D). Furthermore, RNA-seq data indicated that downregulation of all these genes upon TRIM28 knockdown (Fig. 6E). The top 5 motifs for TRIM28 binding sites on proteasome genes were listed (Fig. S3B). Among all these genes encoding proteasome, the protein encoded by PSMB1 is one of the direct targets of BTZ. Therefore, we focused on the regulation of PSMB1 by TRIM28. Chromatin immunoprecipitation followed by qPCR (ChIP-qPCR) assay demonstrated that TRIM28 binds to the promoter region of the PSMB1 gene (Fig. S3C). Knockdown of TRIM28 resulted in a decrease in the mRNA expression levels of PSMB1 (Fig. S3D). Collectively, these findings suggest that TRIM28 could transcriptionally activates the expression of multiple proteasome genes including PSMB1, the direct target of BTZ. Given the crucial roles played by proteasome activity in determining the sensitivity of MM cells to PIs, we explored the regulatory effects of TRIM28 on the sensitivity of MM cells to PIs. The results revealed that TRIM28 OE led to a notable reduction in the sensitivity of MM cells to BTZ and carfilzomib (CFZ), whereas TRIM28 KD enhanced their responsiveness to both drugs (Fig. 6F & Fig. S3E). The in vivo study of TRIM28-OE cells was conducted using a xenograft myeloma murine model. revealing that overexpression of TRIM28 resulted in an increased tumor burden and decreased sensitivity to BTZ treatment (Fig. 6G).

### TRIM28 facilitates ubiquitin-dependent degradation of 14–3 - 3ζ and enhances autophagy in MM

**Fig. 7:**
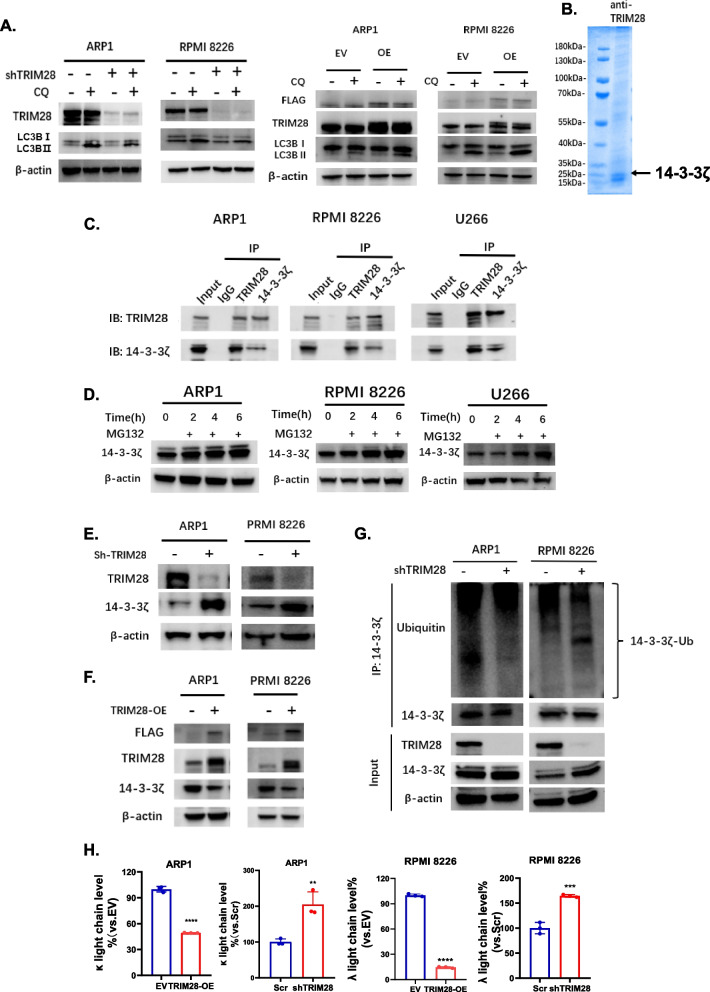
TRIM28 promotes ubiquitin dependent degradation of 14–3 - 3ζ and enhances autophagy in MM cells. **A** Western blots showing the protein levels of LC3BI, LC3BII, and β-actin in MM cell lines ARP1 and RPMI 8226 after overexpressing and knocking down TRIM28 with or without CQ. **B** Proteins binding to TRIM28 were pulled down by anti-TRIM28 antibodies and stained with Coomassie brilliant blue. **C** Endogenous interaction of 14–3 - 3ζ and TRIM28 detected by co-immunoprecipitation and western blot analysis. **D** Western blots showing the expression level of 14–3 - 3ζ in ARP1, RPMI 8226, and U266 cells after treatment with MG132 (10 μM) for indicated times (0 h, 2 h, 4 h, 6 h). The MG132 was used to block the protein degradation process via ubiquitin–proteasome pathway. **E** Western blots showing the protein levels of 14–3 - 3ζ, TRIM28, and β-actin in MM cell lines after TRIM28 KD. **F** Western blots showing the protein levels of 14–3 - 3ζ, TRIM28, and β-actin in MM cell lines after TRIM28 OE. **G** MM cells following TRIM28 KD were lysed and 14–3 - 3ζ was immunoprecipitated by anti- 14–3 - 3ζ antibodies and ubiquitination levels were analyzed by Western blot. **H** Bar plots showing the secreted light chain protein level in MM cell lines ARP1 and RPMI 8226 after overexpressing and knocking down TRIM28 detected by ELISA assay. Data were analyzed using unpaired Student t tests: * *P* < 0.05, ** *P* < 0.01, *** *P* < 0.001, **** P < 0.0001

Next, our intention is to investigate the roles of TRIM28 in activating autophagy in MM cells. Upon inhibiting downstream lysosomal degradation with CQ, we observed a decrease in autophagosome formation in TRIM28-KD cells. Conversely, TRIM28-OE cells exhibited increased autophagosome formation, suggesting that TRIM28 promotes autophagosome formation and activates autophagy (Fig. 7A). It has been reported that TRIM28 exhibits E3 ligase function through its RING domain. Therefore, to elucidate the underlying molecular mechanism by which TRIM28 facilitates autophagy, we opted to examine its interacting partners. Immunoprecipitation-Mass Spectrometry (IP/MS) analysis was performed using anti-TRIM28 antibody in ARP1 cells. Our data indicated that over 264 proteins bound to TRIM28, including 14–3 - 3ζ, a well-known negative regulator of autophagy (Fig. 7B). We utilized co-immunoprecipitation (Co-IP) experiments to confirm the interaction between 14–3 - 3ζ and TRIM28 proteins (Fig. 7C). Additionally, upon treatment with proteasome inhibitor MG132 in MM cells, the expression levels of 14–3 - 3ζ gradually accumulated with prolonged treatment time, suggesting that 14–3 - 3ζ undergoes degradation via the ubiquitin–proteasome pathway (Fig. 7D). Besides, the expression of 14–3 - 3ζ was downregulated in TRIM28-OE cells, whereas in cells with TRIM28-KD, the expression level of 14–3 - 3ζ was upregulated (Fig. 7E & F). Furthermore, a significant decrease in the polyubiquitination level of 14–3 - 3ζ was observed in TRIM28-KD cells, suggesting that TRIM28 plays a critical role in modulating protein levles of 14–3 - 3ζ by regulating its ubiquitination modification (Fig. 7G). It is well-established that 14–3 - 3ζ proteins possess inhibitory functions on autophagy. In this context, TRIM28 may potentially enhance autophagy levels in MM cells by downregulating the expression of 14–3 - 3ζ. Further experiments revealed that TRIM28-KD cells exhibited higher secretion of kappa or lambda light chains, while TRIM28-OE cells displayed lower levels of light chain secretion (Fig. 7H). These findings indicate that TRIM28 could disrupt the balance between protein capacity and protein load, thereby mediating resistance to proteasome inhibitor.

In addition to regulating protein homeostasis, the overexpression of TRIM28 significantly accelerated the proliferation of tumor cells, while knockdown of TRIM28 resulted in a reduction in cell proliferation (Fig. S4A). Furthermore, TRIM28 knockdown led to apoptosis in both ARP1 and RPMI 8226 cells (Fig. S4B). Cell cycle analysis revealed that downregulation of TRIM28 caused cell cycle arrest in G1 phases (Fig. S4C). Given the alterations observed in the DNA damage signaling pathway following TRIM28 knockdown based on RNA-seq data, we conducted further investigations into the potential role of TRIM28 in the DNA damage repair. Experimental results demonstrated an increased level of DNA damage within cells upon TRIM28 KD, characterized by elevated γH2 AX levels similar to those observed after UV irradiation (Fig. S4D). Subsequently, we assessed changes in cellular sensitivity to cytotoxic agents following TRIM28 KD. We observed a substantial increase in sensitivity to Epirubicin (EPI) after TRIM28 KD (Fig. S4E). These findings indicate that beyond its role in regulating protein homeostasis, TRIM28 also modulates various biological processes including cell proliferation, cell cycle progression, apoptosis, and DNA damage repair. Further comprehensive investigation is warranted regarding its function in MM.

## Discussion

Proteostasis refers to the delicate balance of protein synthesis, folding, and degradation in cells [36]. Multiple myeloma is an incurable malignancy of plasma cells in the bone marrow. It is characterized by the secretion of a large number of monclonal immunoglobulins or light chains. Consequently, the exploration of therapeutic agents exhibiting modulatory effects on proteostasis bears profound clinical implications in MM. The proteasome pathway and autophagy pathway are crucial for maintaining proper turnover and cellular homeostasis through protein degradation [36]. PIs have demonstrated paramount significance in the therapeutic management of MM. However, previous research showed that treatment with proteasome inhibitors leads to the accumulation of protein aggregates in the aggresome in MM cells. Subsequently, these aggregates are engulfed by autophagosomes and fuse with lysosomes, culminating in the degradation of the aggregated proteins [37–39]. This process contributes to the maintenance of protein homeostasis and PIs resistance of MM cells [8]. Therefore, inhibitors simultaneously targeting of both autophagy and the proteasomal protein degradation pathway holds significant clinical relevance in multiple myeloma. In this study, we introduced the moiety of an HDAC6 inhibitor into the I3MO structure to synthesize a novel conjugate compound 8b. Our findings revealed that 8b exhibited stronger cytotoxic effects on MM cell lines and primary cells from patients compared to treatment with I3MO or HDAC6i alone. Most importantly, our synthesized compound 8b effectively reduced proteasome activity and suppressed aggresome and autophagosome formation. Combining 8b with BTZ inhibited BTZ-induced pro-survival autophagy, thereby enhancing cytotoxic effect of BTZ. These findings highlight the therapeutic potential of 8b in overcoming MM drug resistance by targeting aggresome and autophagosome pathways.

Through RNA-seq analysis, we identified that treatment with 8b significantly downregulated TRIM28 promoter activity and subsequently reduced TRIM28 expression at both mRNA and protein levels. This is the first time TRIM28 function has been associated with proteasome activity and autophagy regulation in MM cells while its specific role in myelomagenesis remains not fully understood. Although little is known about the roles of TRIM28 in MM, its expression is upregulated in high-risk MM patients [40]. Studies have found that abnormally expression of TRIM28 in gastric cancer, colorectal cancer, and non-small cell lung cancer, indicating its crucial roles in various kind of cancers [41–43]. In our results, we also have observed highly expression level of TRIM28 in MM patients, promoting MM cell survival, proliferation and drug resistance. TRIM28 functions as a chromatin-associated protein and transcriptional regulator [29, 44]. Emerging evidence suggests that TRIM28 exerts a broad influence on gene expression by modulating chromatin structure and transcriptional complexes. As a transcriptional coregulator, TRIM28 is essential for the regulatory function of KRAB-ZNF proteins, which recognize specific DNA motifs via their zinc finger domains and recruit TRIM28 to these loci. Acting as a scaffold, TRIM28 facilitates the assembly of heterochromatin-inducing complexes, including SETDB1 and the NuRD complex, leading to histone deacetylation. Additionally, TRIM28 interacts with HP1 proteins via the PxVxL motif, reinforcing heterochromatin stability by associating with the H3 K9 me3 mark [45]. Beyond chromatin remodeling, TRIM28 also plays a crucial role in stabilizing RNA polymerase II (Pol II) pausing at transcriptional start sites, with its phosphorylation status at Ser824 serving as a key determinant of Pol II activity. Phosphorylated TRIM28 facilitates Pol II release from pausing, enabling rapid transcriptional activation. Collectively, these findings underscore TRIM28 as a master regulator of gene expression, integrating chromatin modifications and Pol II dynamics to control transcriptional output, with significant implications for cellular function and disease pathogenesis [46–48]. Zhang et al. reported that TRIM28 activates the expression of PSMB2 by entering the nucleus, enhancing proteasome activity and mediating bortezomib resistance in HCC [30]. Our study showed that TRIM28 locates in the nucleus of MM cells and binds to the promoter region of PSMB1 validated by both ChIP-seq and ChIP-qPCR. Knockdown of TRIM28 resulted in downregulation of PSMB1, indicating its role as a transcriptional regulator involved in the regulation of proteasome subunits PSMB1. Interestingly, the ChIP-seq data showed that TRIM28 binds to multiple genes encoding proteasome subunits, which was confirmed by RNA-seq data showing their downregulation upon TRIM28 knockdown. These genes included PSMB1, PSMB4, PSMB8, PSMB9, PSMD2, PSMD4, PSMD9 and PSMD13. Among these subunits related to proteasome function and important for various cancer biological function [49–53], it has been reported that PSMD4 plays a critical role as a survival gene in multiple myeloma [54]. Therefore, further investigations are needed to explore the regulatory role of TRIM28 on these genes encoding proteasome subunits. This will provide a more comprehensive understanding of how TRIM28 regulates the expression of different proteasome subunits and shed light on its broader role in the cellular machinery involved in protein degradation.

In addition to its role as a transcription regulator, TRIM28 also regulates autophagy by promoting the SUMOylation modification of its substrates. Yang et al. discovered that TRIM28 mediates Lys840 SUMOylation, which enhances Vps34 activity and binds to Beclin1, thereby facilitating autophagosome formation [55]. Our research reveals the regulatory role of TRIM28 in autophagy in MM for the first time. TRIM28 can influence autophagy by mediating the ubiquitination and degradation of 14–3 - 3ζ through its E3 ubiquitin ligase activity. 14–3 - 3ζ is an important member of the 14–3 - 3protein family, which plays a crucial role in maintaining cellular homeostasis. These proteins interact with over 1200 protein binding partners, typically through phosphorylation-dependent mechanisms to intricately regulate various aspects of cellular function including localization, posttranslational modifications, biomolecular interactions, and enzymatic or transcriptional activities of their partner proteins [56, 57]_._ The 14–3 - 3ζ acts as a negative regulator of autophagy by inhibiting the Vps34 kinase activity, which is crucial for autophagosome membrane assembly in the early stages of autophagy [58]. Additionally, it binds to phosphorylated TFEB, a transcriptional factor of multiple autophagy-related genes, and retains it in the cytosol, thereby inhibiting autophagosome and autophagic formation and flux [59, 60]. Our research suggests that TRIM28 may regulate autophagy in MM by controlling the stability of 14–3 - 3ζ proteins. This insight into the interplay between TRIM28 and 14–3 - 3ζ sheds light on the complex regulatory mechanisms governing autophagy in MM. Interestingly, 14–3 - 3ζ also limits proteasome assembly and cellular capacity for protein degradation while conferring resistance to proteasome inhibitors in MM [61, 62]^.^ Further research is needed to explore whether TRIM28 partially mediate proteasome inhibitor effects through regulating the stability of 14–3 - 3ζ and promoting proteasome assembly. TRIM28, functioning as an E3 ubiquitin ligase, has been reported to regulate the stability of multiple key proteins involved in cancer-related biological processes, including p53, RB, RLIM, and p27. Among these substrates, p53 and RB play particularly crucial roles in MM tumor suppression. TRIM28 has been reported to interact with MDM2, enhancing p53 ubiquitination and degradation. Wang et al. demonstrated that TRIM28 cooperates with MDM2 to promote p53 degradation, while Liu et al. showed that MAGE proteins can inhibit p53 ubiquitination by binding to MDM2 [63, 64]. Interestingly, TRIM28 overexpression competes with MAGE proteins, thereby facilitating p53 degradation and promoting tumor progression [64]. Additionally, in lung cancer, RLIM has been found to interact with MDM2, promoting its degradation, while TRIM28 enhances RLIM ubiquitination, ultimately leading to reduced p53 expression [23, 25, 48]. In our study, TRIM28 KD induced DNA damage response within cells, a well-established downstream function of p53, characterized by elevated γH2 AX levels. As an E3 ligase, TRIM28 has been shown to interact with CDK4/6-phosphorylated RB1, promoting its ubiquitination and degradation, thereby facilitating tumor cell survival. In our study, TRIM28 knockdown resulted in reduced cell proliferation, cell cycle arrest, and activation of the DNA damage response—findings consistent with the tumor-suppressive function of RB1. However, the precise molecular relationship between TRIM28 and RB1 in MM remains to be fully elucidated. Given the critical role of TRIM28 in regulating both p53 and RB1, further exploration of its substrate proteins as an E3 ligase in MM is essential. Such studies could provide key insights into TRIM28 as a potential therapeutic target in MM.

## Conclusion

In summary, our study demonstrated that the novel I3MO derivative 8b effectively induces proteasome and autophagy inhibition, presenting a promising therapeutic strategy to improve patient outcomes in MM. Furthermore, our findings unveiled the pivotal role of TRIM28, targeted by 8b treatment, in maintaining protein homeostasis in MM by activating both the proteasome and autophagy pathways, positioning it as a potential therapeutic target.

## Supplementary Information

Supplementary Material 1.

## Data Availability

The datasets generated during and/or analysed during the current study are available from the corresponding author on reasonable request.
