# Supplementary material for: A novel indirubin- 3-monoxime derivative I3MV- 8b exhibits remarkable cytotoxicity against multiple myeloma by targeting TRIM28
Source: Biomark Res. 2025 Apr 7;13:57. doi: 10.1186/s40364-025-00773-3 (PMC11978164; doi:10.1186/s40364-025-00773-3)
Supplement: Supplementary file 1 — Supplementary Material 1. [file 40364_2025_773_MOESM1_ESM.pdf]

Figure S1.

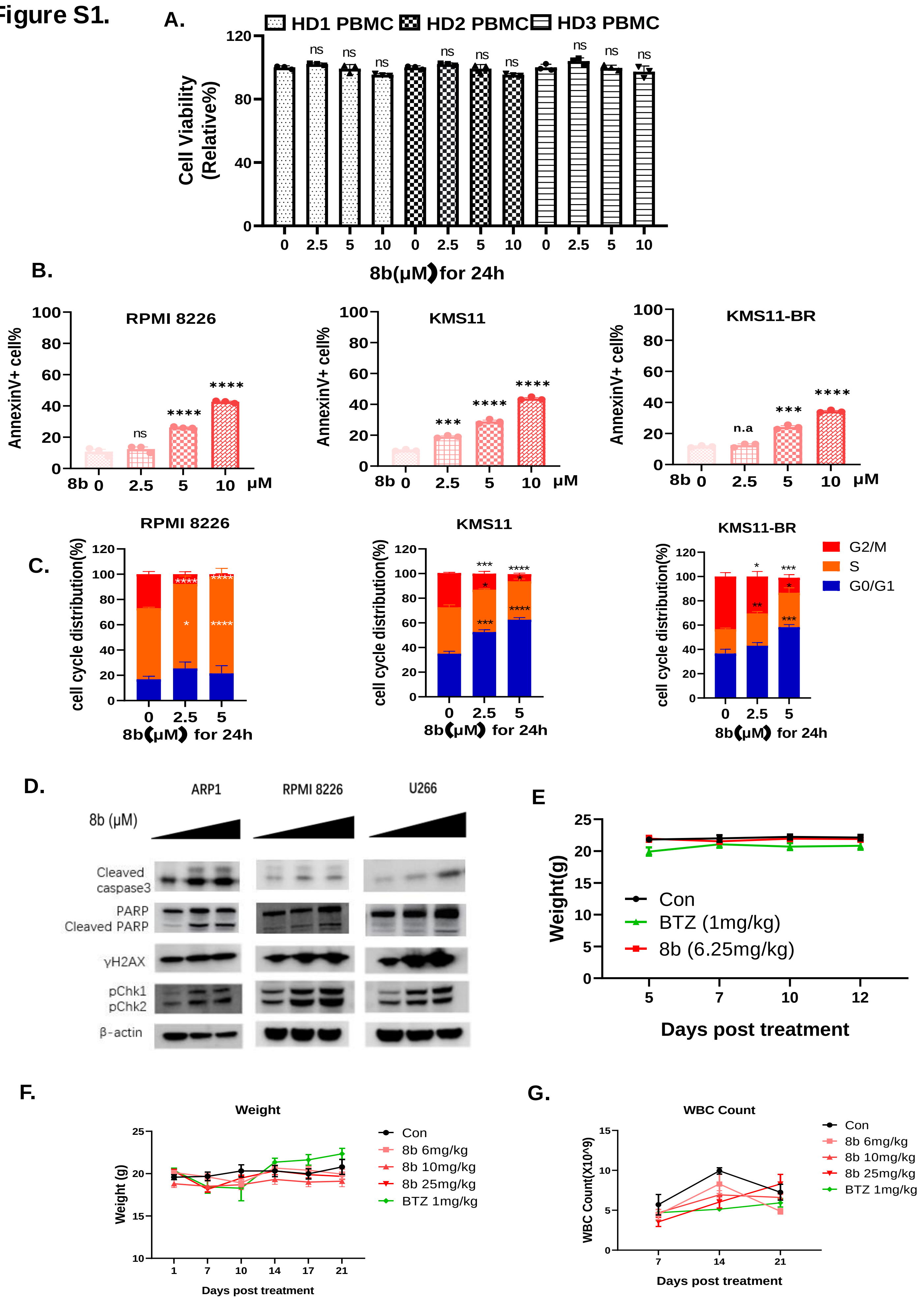
A.
HD1 PBMC
HD2 PBMC
HD3 PBMC
Cell Viability (Relative%)
8b(μM) for 24h
B.
RPMI 8226
KMS11
KMS11-BR
AnnexinV+ cell%
8b 0 2.5 5 10 μM
C.
cell cycle distribution(%)
G2/M
S
G0/G1
8b (μM) for 24h
D.
ARP1
RPMI 8226
U266
8b (μM)
Cleaved caspase3
PARP
Cleaved PARP
γH2AX
pChk1
pChk2
β-actin
E
Weight(g)
Con
BTZ (1mg/kg)
8b (6.25mg/kg)
Days post treatment
F.
Weight
Weight (g)
Con
8b 6mg/kg
8b 10mg/kg
8b 25mg/kg
BTZ 1mg/kg
Days post treatment
G.
WBC Count
WBC Count(X10^9)
Con
8b 6mg/kg
8b 10mg/kg
8b 25mg/kg
BTZ 1mg/kg
Days post treatment

Figure S2.

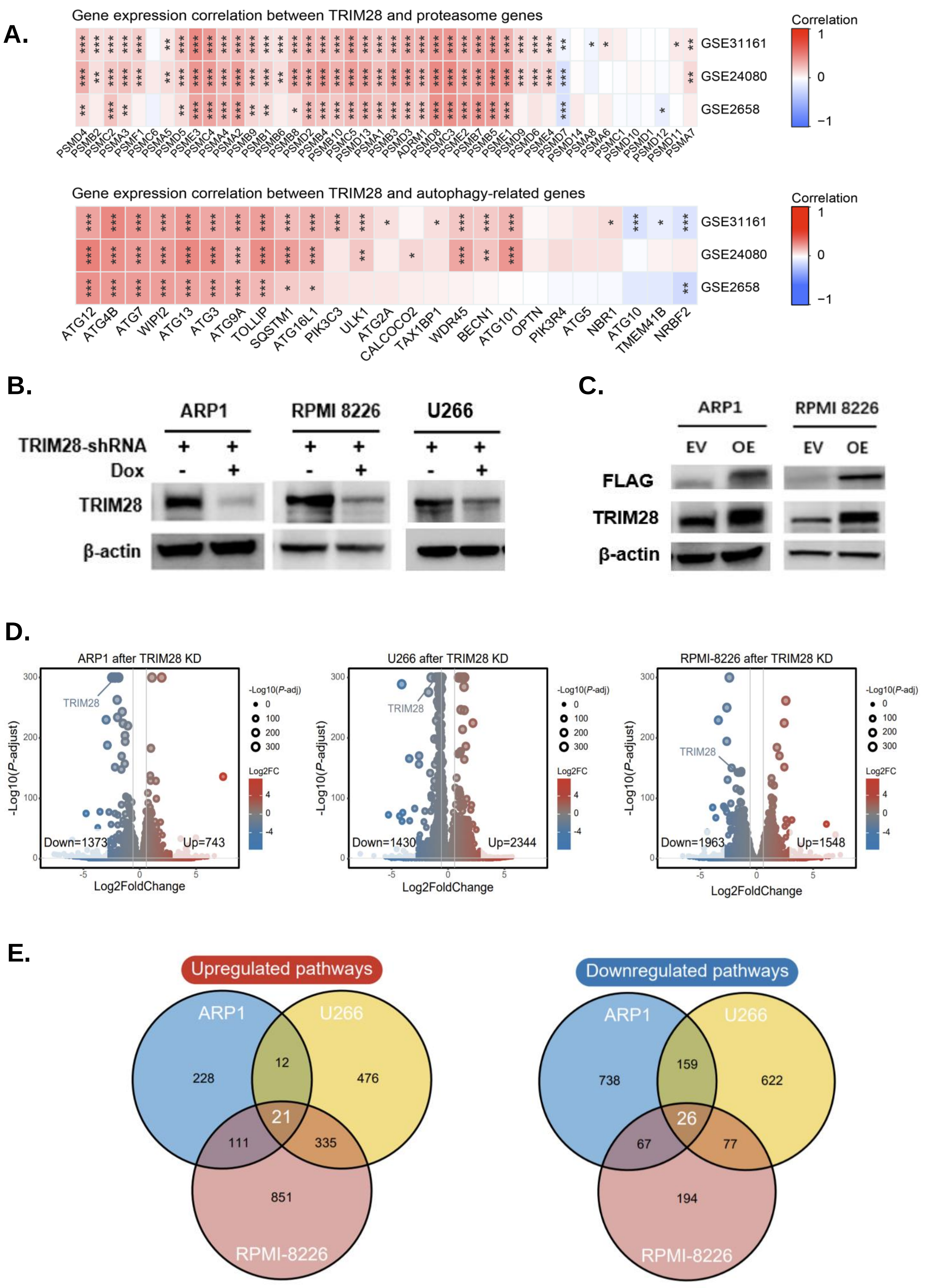

A.
Gene expression correlation between TRIM28 and proteasome genes
GSE31161
GSE24080
GSE2658
Correlation
1
0
−1
PSMD4 PSMB2 PSMC2 PSMA3 PSMF1 PSMC6 PSMA5 PSMD5 PSME3 PSMC4 PSMA4 PSMA2 PSMB9 PSMB1 PSMB6 PSMB8 PSMD2 PSMB4 PSMB10 PSMC5 PSMD13 PSMA1 PSMB3 PSMD3 ADRM1 PSMD8 PSMC3 PSME2 PSMB7 PSMB5 PSME1 PSMD9 PSMD6 PSME4 PSMD7 PSMD14 PSMA8 PSMA6 PSMC1 PSMD10 PSMD1 PSMD12 PSMD11 PSMA7
Gene expression correlation between TRIM28 and autophagy-related genes
GSE31161
GSE24080
GSE2658
Correlation
1
0
−1
ATG12 ATG4B ATG7 WIPI2 ATG13 ATG3 ATG9A TOLLIP SQSTM1 ATG16L1 PIK3C3 ULK1 ATG2A CALCOCO2 TAX1BP1 WDR45 BECN1 ATG101 OPTN PIK3R4 ATG5 NBR1 ATG10 TMEM41B NRBF2
B.
ARP1
RPMI 8226
U266
TRIM28-shRNA
Dox
TRIM28
β-actin
C.
ARP1
RPMI 8226
EV
OE
FLAG
TRIM28
β-actin
D.
ARP1 after TRIM28 KD
U266 after TRIM28 KD
RPMI-8226 after TRIM28 KD
-Log10(P-adjust)
Log2FoldChange
TRIM28
Down=1373
Up=743
Down=1430
Up=2344
Down=1963
Up=1548
-Log10(P-adj)
0
100
200
300
Log2FC
E.
Upregulated pathways
ARP1
U266
RPMI-8226
228
12
476
21
111
335
851
Downregulated pathways
ARP1
U266
RPMI-8226
738
159
622
26
67
77
194

Figure S3.

A.

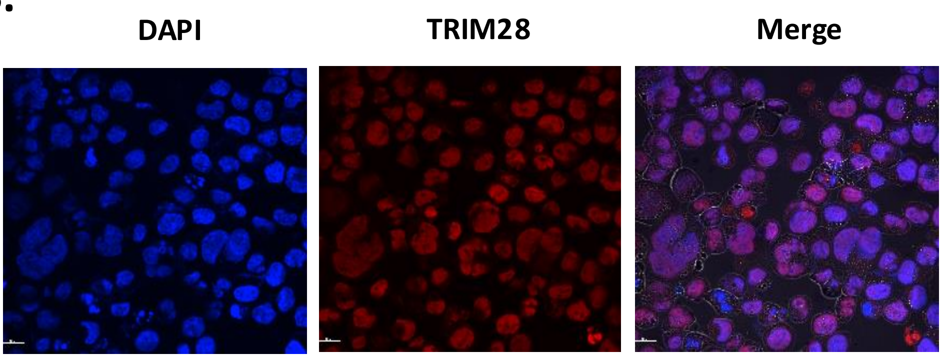

B.

TOP5 motifs for TRIM28 binding sites of proteasome genes

Rank	Motif	*P*-value	% of Targets
1	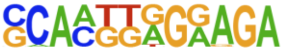	1e-7	50.00%
2	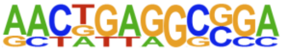	1e-7	50.00%
3	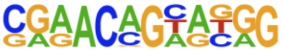	1e-6	50.00%
4	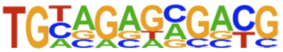	1e-5	62.50%
5	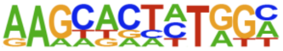	1e-5	50.00%

C.

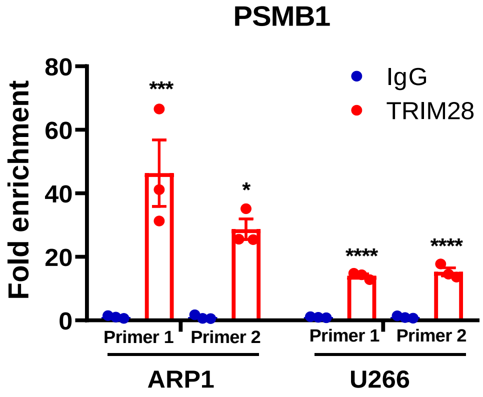

D.

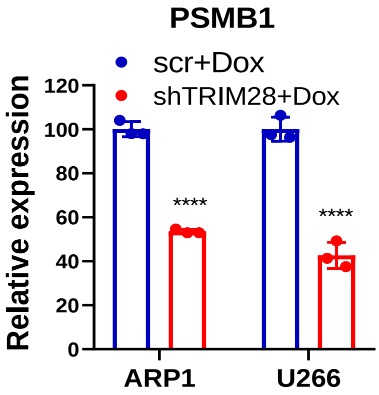

E.

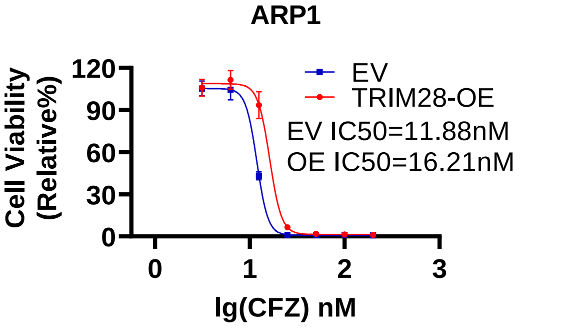

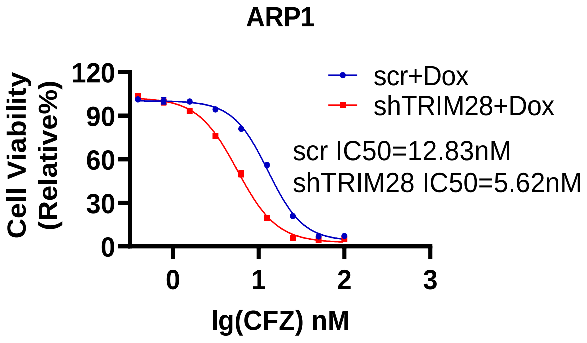

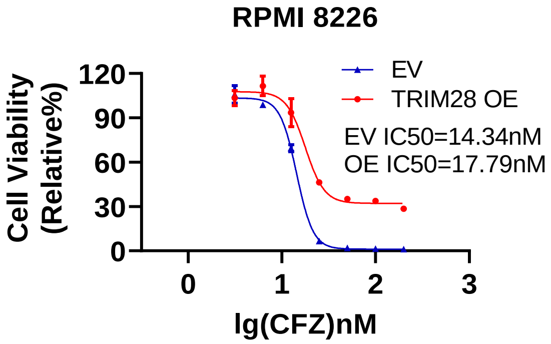

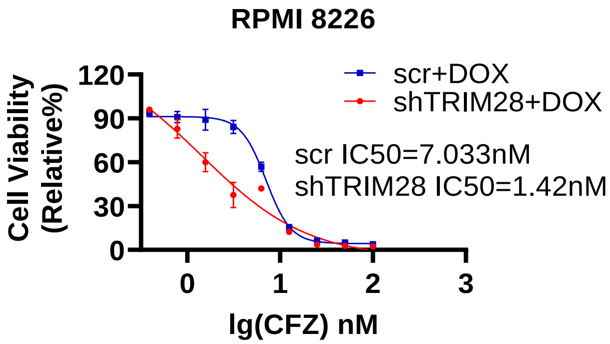

Figure S4.

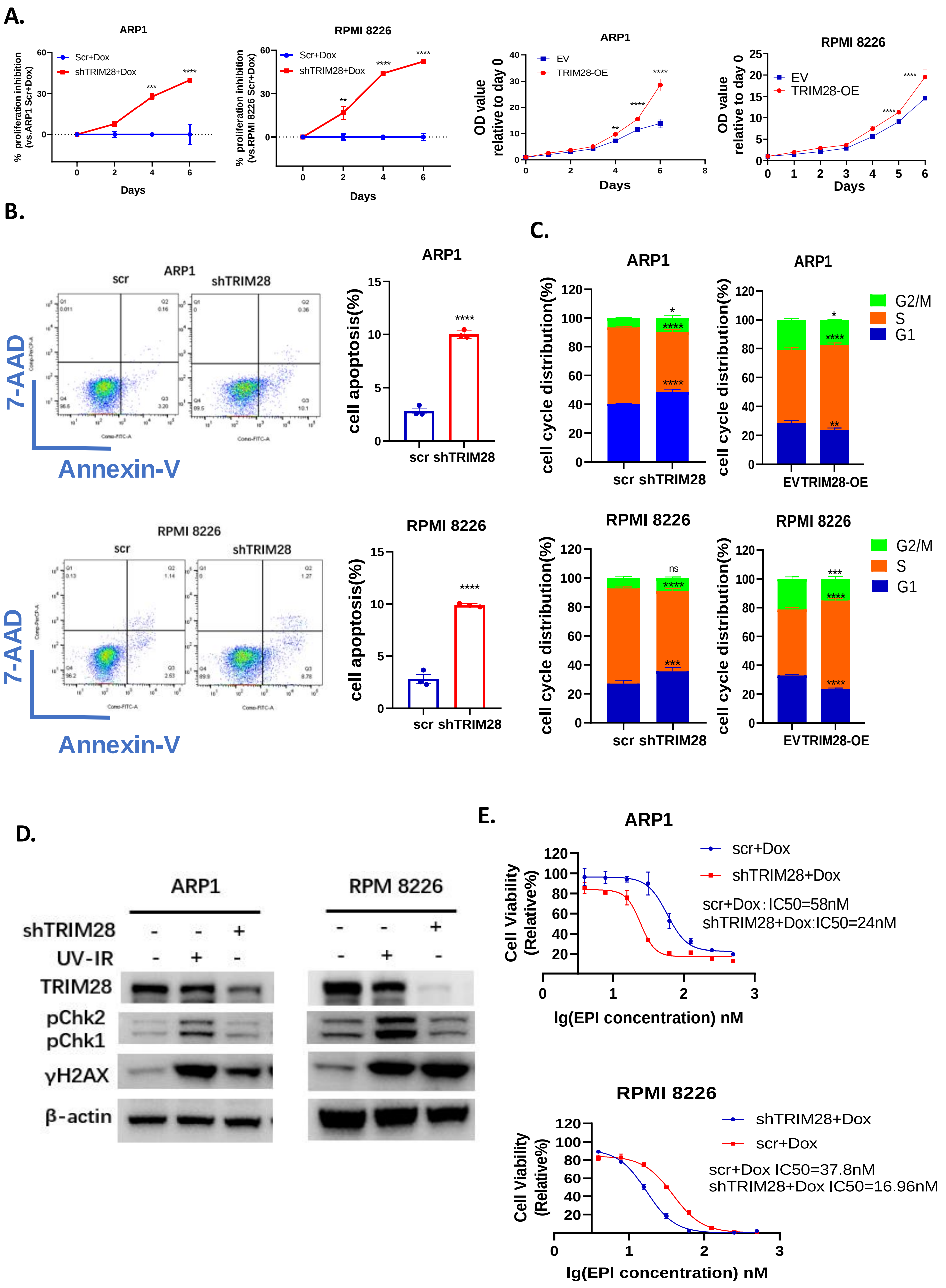

A.
ARP1
Scr+Dox
shTRIM28+Dox
% proliferation inhibition (vs.ARP1 Scr+Dox)
Days
RPMI 8226
% proliferation inhibition (vs.RPMI 8226 Scr+Dox)
ARP1
EV
TRIM28-OE
OD value relative to day 0
Days
RPMI 8226
B.
ARP1
scr
shTRIM28
7-AAD
Annexin-V
cell apoptosis(%)
scr shTRIM28
RPMI 8226
C.
ARP1
cell cycle distribution(%)
G2/M
S
G1
EVTRIM28-OE
RPMI 8226
D.
ARP1
RPM 8226
shTRIM28
UV-IR
TRIM28
pChk2
pChk1
γH2AX
β-actin
E.
ARP1
scr+Dox
shTRIM28+Dox
scr+Dox : IC50=58nM
shTRIM28+Dox:IC50=24nM
Cell Viability (Relative%)
lg(EPI concentration) nM
RPMI 8226
shTRIM28+Dox
scr+Dox
scr+Dox IC50=37.8nM
shTRIM28+Dox IC50=16.96nM

Supplementary figure

Figure S1.

A. Bar plots showing different cytotoxic effect of 8b on Peripheral blood mononuclear cell (PBMC) of bone marrow aspirates from 3 healthy donors (HD).

B. Statistical bar plots showing the percentage of apoptotic cells in MM cell line RPMI 8226, KMS11, KMS11-BR detected by flow cytometry after treatment of DMSO control, 2.5μM, 5μM 8b, 10μM 8b for 24 hours. The data are triplets and shown as mean ± SEM. * P < 0.05, ** P < 0.01, *** P < 0.001, **** P < 0.0001 (one-way ANOVA test).

C. Bar plots showing cell cycle distribution in MM cell lines RPMI 8226, KMS11, KMS11-BR detected by flow cytometry after treatment of DMSO control, 2.5 μM and 5 μM 8b for 24 hours. The results are presented as percentage of cells in each phase of the cell cycle (G0/G1, S, and G2/M) calculated by Flow jo software.

D. Western blots showing the protein levels of cleaved caspase-3, cleaved PARP, γH2AX, pChk1, pChk2, and β-actin in MM cell lines ARP1, RPMI 8226, and U266 after treatment with DMSO control or increasing concentrations of 8b (5μM, and 10μM) for 24 hours.

E. Line chart showing the changes in body weight in mice bearing MM xenografts under the treatment of DMSO control, 6.25 mg/kg 8b, or 1 mg/kg BTZ.

F. Line chart showing the changes in body weight over time in mice under the treatment of DMSO control, 6 mg/kg 8b, 10 mg/kg 8b, 25 mg/kg 8b, or 1 mg/kg BTZ.

G. Line chart showing the changes in white blood cells count over time in mice under the treatment of DMSO control, 6 mg/kg 8b, 10 mg/kg 8b, 25 mg/kg 8b, or 1 mg/kg BTZ. The data are triplets and shown as mean ± SEM. * P < 0.05, ** P < 0.01, *** P < 0.001, **** P < 0.0001

Figure S2.

A. (Upper) Heatmap displaying the correlation between multiple proteasome-encoding genes and the expression levels of TRIM28 in various MM RNA-seq dataset. (Lower) Heatmap displaying the correlation between multiple autophagy-encoding genes and the expression levels of TRIM28 in various MM RNA-seq dataset.

B. MM cells transfected with shTRIM28 plasmid were treated with 1 μg/ml Doxycycline for 48 hours and lysed. Western blots showing protein level of TRIM28 and β-actin.

C. MM cells transfected with TRIM28 overexpressing plasmid or empty vector (EV) were lysed. Western blots showing protein level of FLAG, TRIM28 and β-actin.

D. The volcano plot illustrating the number of genes upregulated and downregulated in three cell lines - ARP1, RPMI 8226, and U266 - following TRIM28 KD.

E. Venn plot illustrating the extent of overlap in the upregulated and downregulated pathways across distinct cell lines following the knockdown of TRIM28.

Figure S3.

A. Subcellular localization of TRIM28 was observed under confocal microscope. ARP1 cells were stained with anti-TRIM28- antibody/Alexa Fluor™ 647 secondary antibody (shown in red). Nuclei were stained with DAPI (blue). Images were acquired digitally by FV1000-X81 confocal microscope (Olympus, Japan) with 60× magnification.

B. Table showing Top5 motifs for TRIM28 binding sites of proteasome genes.
C. RT-qPCR showing PSMB1 mRNA level in TRIM28-KD MM cells. Data represent the mean ± SEM. Statistical significance was assessed using two-tailed Student's t-tests. * $P < 0.05$, ** $P < 0.01$, *** $P < 0.001$, **** $P < 0.0001$.
D. ChIP-qPCR was performed in ARP1 and U266 myeloma cells using anti-TRIM28 antibodies. The DNA occupancy of the PSMB1 promoter was analyzed by qPCR. Data represent the mean ± SEM. Statistical significance was assessed using two-tailed Student's t-tests. * $P < 0.05$, ** $P < 0.01$, *** $P < 0.001$, **** $P < 0.0001$.
E. The dose-response curves of MM cell lines to CFZ after overexpressing and knocking down TRIM28 were plotted based on the viability assay data. Cell viability was assessed by CCK8 after 24 hours of treatment, and IC50 was calculated with GraphPad Prism 8.

Figure S4.

A-C. Effects of TRIM28 on cell proliferation, apoptosis, and cell cycle were detected by CCK-8 and flow cytometry, respectively. All results were presented as means ± SEM of three independent experiments. Statistical significance was assessed using two-tailed Student's t-tests or two-way ANOVA test. * $P < 0.05$, ** $P < 0.01$, *** $P < 0.001$, **** $P < 0.0001$.

D. Western blots showing the expression level of pChk2, pChk1 and γH2AX cells after treatment with UV for 1 hours or TRIM28 KD. in MM cell lines

E. The dose-response curves of MM cell lines after TRIM28 KD to Epirubicin (EPI) were plotted based on the viability assay data. Cell viability was assessed by CCK8 after 48 hours of treatment, and IC50 was calculated with GraphPad Prism 8.
